# Qualitative real-time analysis by nurses of sublingual microcirculation in intensive care unit: the MICRONURSE study

**DOI:** 10.1186/s13054-015-1106-3

**Published:** 2015-11-06

**Authors:** Sébastien Tanaka, Anatole Harrois, Camille Nicolaï, Mélanie Flores, Sophie Hamada, Eric Vicaut, Jacques Duranteau

**Affiliations:** AP-HP, Service d’Anesthésie-Réanimation, Hôpitaux Universitaires Paris-Sud, Université Paris-Sud, Hôpital de Bicêtre, Le Kremlin-Bicêtre, France; Laboratoire d’Etude de la Microcirculation, “Bio-CANVAS: Biomarqueurs in CardiaNeuroVascular Diseases” UMRS 942, Paris, France; Department of Anesthesia and Intensive Care, Bicêtre Hospital, Hôpitaux universitaires Paris-Sud, Université Paris-Sud, Assistance Publique - Hôpitaux de Paris, 78, rue du Général Leclerc, 94275 Le Kremlin Bicêtre, France

## Abstract

**Introduction:**

We aimed to determine i) the feasibility of nurses taking bedside measurements of microcirculatory parameters in real time in intensive care patients; and ii) whether such measurements would be comparable to those obtained by the classical delayed semi quantitative analysis made by a physician.

**Methods:**

This prospective observational study was conducted in a university hospital and was approved by our local Institutional Review Board (IRB 00006477). After ICU admission and study inclusion, a set of measurements of macrocirculatory and microcirculatory parameters was taken by the nurse in charge of the patient every 4 h within the first 12 h after admission and before and after every hemodynamic therapeutic intervention. Seventy-four sublingual microvascular measurements were performed with incident dark field illumination (IDF) microscopy in 20 mechanically ventilated patients hospitalized in the ICU.

**Results:**

There were no significant differences between the microvascular flow index (MFI) taken in real time by the nurses and the delayed evaluation by the physician. In fact, the nurses’ real-time measurement of MFI demonstrated good agreement with the physician’s delayed measurement. The mean difference between the two MFIs was −0.15, SD = 0.28. The nurses’ real-time MFI assessment showed 97 % sensitivity (95 % CI: 84–99 %) and 95 % specificity (95 % CI: 84–99 %) at detecting a MFI <2.5 obtained by a physician upon delayed semiquantitative measurement. Concerning the density, 81 % of the paramedical qualitative density measurements corresponded with the automatized total vessel density (TVD) measurements. The nurses’ real-time TVD assessment showed 77 % sensitivity (95 % CI: 46–95 %) and 100 % specificity (95 % CI: 89–100 %) at detecting a TVD <8 mm/mm^2^.

**Conclusion:**

A real-time qualitative bedside evaluation of MFI by nurses showed good agreement with the conventional delayed analysis by physicians. The bedside evaluations of MFI and TVD were highly sensitive and specific for detecting impaired microvascular flow and low capillary density. These results suggest that this real-time technique could become part of ICU nurse routine surveillance and be implemented in algorithms for hemodynamic resuscitation in future clinical trials and regular practice. These results are an essential step to demonstrate whether these real-time measurements have a clinical impact in the management of ICU patients.

## Introduction

The aim of hemodynamic resuscitation including fluid challenge or vasopressor administration is to restore microcirculation perfusion and tissue oxygenation, to avoid hypoxia, and to protect organ function [1]. Much evidence supports microcirculation playing a major role in the pathogenesis of shock and in the development of organ failure in critically ill patients [1–3]. Persisting alteration of microcirculation despite restoration of macrocirculation is linked to the outcome of patients with shock [4].

The microcirculatory dysfunction is characterized by heterogeneous abnormalities in blood flow perfusion and capillary density [5]. Restoring these capillary abnormalities by targeting not only the macrocirculation but also the microcirculation could become the gold standard in the future [6–8]. However, hemodynamic optimization in the intensive care unit (ICU) is only titrated on macrovascular parameters and no microvascular evaluation method is currently available for routine daily bedside monitoring. There is thus a crucial need to validate a technique able to give pertinent data that may guide the hemodynamic status optimization performed by physicians. To achieve this, the technique must provide physiologically relevant parameters that play a central role and can thus be used as markers of severity and outcome, based on which the hemodynamic strategy could be titrated. It must also be easy to use and provide readily interpretable data to ensure its adoption by all medical and paramedical staff and its inclusion within the global strategy for monitoring hemodynamics in ICU patients.

Using the sidestream dark field (SDF) or incident dark-field illumination (IDF) imaging technologies to assess sublingual microcirculation, several studies have reported a link between microvascular flow index (MFI) and the proportion of perfused vessels (PPV) and outcome [1, 4, 9, 10]. In one such study using a large database of patients with severe sepsis who had undergone evaluation of microvascular perfusion, De Backer et al. recently reported that PPV was a stronger independent predictor of mortality [4]. Their results suggest that these imaging techniques might provide pertinent parameters to guide the hemodynamic management of ICU patients and to prevent organ dysfunction. However, the microvascular measurements were semiquantitative and required time to analyze videos with use of software, thus preventing their real-time utilization to titrate necessary hemodynamic support [11, 12]. Before implementing these microcirculatory parameters in algorithms for hemodynamic resuscitation, we first needed to ensure the feasibility of monitoring and analyzing sublingual microcirculation in real time in daily practice. Thus the present study was designed to answer the following questions.

1. Can microcirculatory parameters be measured in real time by nurses at the bedside in intensive care patients?
2. If they can, are these measurements comparable with those obtained by the classical delayed semiquantitative analysis made by a physician?

## Methods

### Patients

This prospective observational study was conducted in a university hospital and was approved by our local Institutional Review Board (Comité pour la protection des personnes IledeFrance VII; IRB 00006477) and informed consent had been obtained from the patients or their relatives. Patients were recruited in the 28-bed surgical intensive care unit of Bicêtre University Hospital between August 2014 and October 2014.

Adult patients admitted to the ICU were enrolled in this prospective observational study when they were sedated and mechanically ventilated and had been equipped with an arterial catheter and a cardiac output monitor (PiCCO 2 device (Pulsion Medical Systems®, Munich, Germany)). The criteria for exclusion from the study were age <18 years, pregnancy, maxillofacial trauma and oral mucosal injuries hampering the performing of sublingual microcirculation monitoring.

## Study design and measurements

After ICU admission and study inclusion, the nurse in charge of the patient performed a set of measurements of macrocirculatory and microcirculatory parameters every 4 h within the first 12 h after admission and before and after every hemodynamic therapeutic intervention, such as fluid challenge, transfusion of red blood cells (RBC) or change in catecholamine rate during this period. Macro hemodynamic data included heart rate, mean arterial pressure, delta pulse pressure (ΔPP) and cardiac index. Sublingual microcirculatory measurements were performed with an incident dark-field illumination device (Cytocam-IDF (incident dark-field illumination), Braedius Medical, Huizen, The Netherlands). Lactate concentration, hemoglobin concentration, norepinephrine or epinephrine dose during the procedure were gathered. The simplified acute physiology score II (SAPSII) at admission and the sequential organ failure assessment (SOFA) score were calculated at the inclusion of each patient.

## Microvideoscopic measurements and analysis

The new technology Cytocam-IDF imaging device consists of a pen-like probe incorporating IDF illumination with a set of high-resolution lenses projecting images on to a computer-controlled image sensor synchronized with a short-pulsed illumination light. This technology was validated in a recent study that compared the Cytocam-IDF device with the SDF technology [13]. Flow characteristics of the microvasculature were quantified using the MFI, a semiquantitative technique consistent with recommendations from a consensus conference on microcirculatory image analysis in human subjects [14]. The image is divided into four quadrants and the vessels <20 μm diameter are assigned a score based on the predominant flow characteristics of the vessels in that quadrant (0 = absent flow; 1 = intermittent; 2 = sluggish; 3 = normal). The values in each quadrant are averaged to give an MFI for each sublingual site at each time point [14]. A quantitative measurement of the capillary density, the total vessel density (TVD, mm/mm^2^), was also taken automatically with dedicated software (Cytocam video microscope, Braedius®, Netherlands).

Each set of measurements was performed at the bedside in real time by the nurse in charge of the patient. The nurse calculated the MFI score (nurses’ real-time MFI) once a 5-s video sequence had been obtained. For assessment of density, the nurse qualitatively evaluated the type of density as poor, normal or rich (nurses’ real-time TVD). Videos initially analyzed by the nurses were associated to an alphanumeric code to enable offline and delayed analysis (physician’s delayed MFI and TVD) by a physician experienced in Cytocam-IDF analysis (ST) blinded to all clinical data and nurses’ real-time MFI values.

## Nurse training

Sixteen nurses were trained to use the device before the beginning of the study. All the nurses passed the training process. During the nurse training, we followed the guidelines for optimal image acquisition and analysis of microcirculation [14]. Briefly, the nurses underwent 90 minutes of didactic theoretical training and a minimum of 60 minutes hands-on training. This training was performed by a physician from the intensive care unit (ST). To prevent the pressure artifact, which is a main concern, it was recommended that the microscope be pulled back gently until contact is lost and then to advance the probe again slowly to the point at which contact is regained [14]. In order to be sure of the technical quality of the videos, the physician in charge of the training ensured that the quality of the videos made by the nurses was good.

**Fig. 1:**
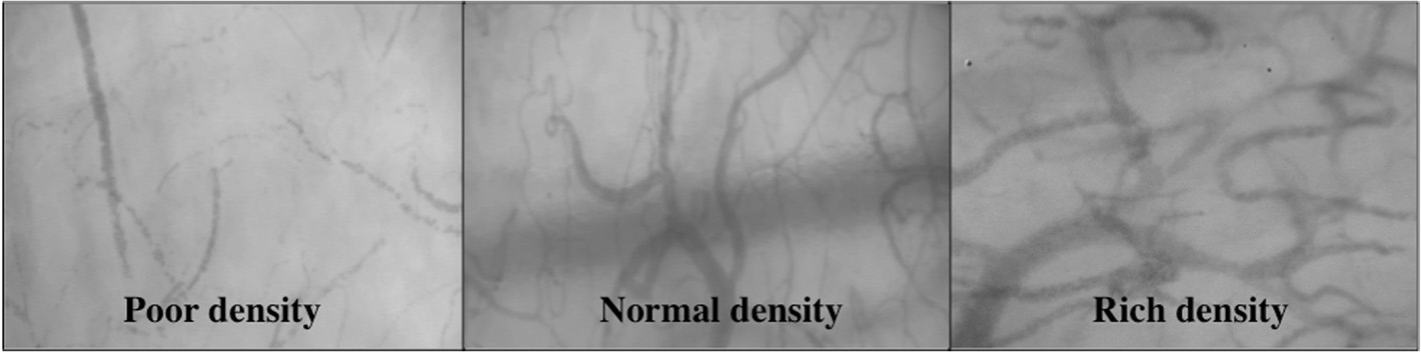
Different types of microvascular density (poor, normal and rich)

After the theoretical training, the nurse and the physician compared the quality of videos successively obtained. No nurse has been authorized to take part in the study until the physician in charge of the training considered that the quality of the videos was good. We also agree that pressure artifact is a main concern. The quality of the nurses’ videos has been analyzed by the Microcirculation Image Quality Score developed by Massey et al. [15]. Six categories (illumination, duration, focus, content, stability, and pressure) were used for scoring each video clip. Each category was scored as optimal (0 points), suboptimal but acceptable (1 point), or unacceptable(10 points). Massey et al. consider that any video with a cumulative score of 10 or higher is unacceptable for further analysis [15]. To assess flow, the nurses learned to divide the screen into four quadrants in order to quantify in real time the major flow type in each quadrant and calculate the MFI score. By convention, capillary density was considered poor for TVD <8 mm/mm^2^, normal for 8 ≤ TVD ≤12 mm/mm^2^ and rich for TVD >12 mm/mm^2^. Videos and photos showing different types of density (poor, normal and rich) were shown and explained to each nurse (Fig. 1).

### Statistical analysis

As most microcirculatory variables have a non-Gaussian distribution, the non-parametric Mann–Whitney test was used. Values are presented as the median (25th–75^th^ percentile). Categorical variables are expressed as a number (%).

Correlation between nurses’ real-time MFI and the physician’s delayed MFI was assessed using non-parametric Spearman‘s correlation. The agreement between these two MFI measurements was tested using the Bland–Altman method [16]. The physician’s delayed MFI served as the reference standard for this study. The sample size estimate for the Bland–Altman method was based on 95 % confidence intervals around the standard deviation (SD) of the difference between the two measurements. As calculated by Bland and Altman the standard error of the 95 % limit of agreement is approximately root (3 s^2^/n), where s is the standard deviation of the differences between measurements by the two methods and n is the sample size. Thus, we considered that if the limit of agreement given by d-2 s is around 0.5, a sample size of 75 would allow precision in the estimate of the limit of agreement around ± 0.05. The sensitivity and the specificity of the nurses’ real-time MFI and TVD to respectively detect impaired microvascular flow and low microvascular density were calculated using the physician’s delayed MFI and TVD as the reference standard. An impaired microcirculatory flow was defined as an MFI <2.5. A low TVD was defined as a TVD <8 mm/mm^2^. In order to test the potential heterogeneous performances between nurses, we calculated the delta of MFI measured by nurses and by the physician and used one-way analysis of variance (ANOVA) to compare the nurses. The data were analyzed using Prism, San Diego, California 4® for Mac OS X®. A *p* value <0.05 was considered to be statistically significant.

## Results

Twenty patients were enrolled in the study. The main characteristics of these patients are presented in Table 1. Table 2 provides hemodynamic data of patients. Seventy-four microvascular sets were performed by nurses (3.7 ± 1.0 mean ± SD measurements/patient). After acquisition of a video, the bedside analysis by the nurses of real-time MFI and TVD required less than 3 minutes. For technical quality of the nurses’ videos, the Microcirculation Image Quality Score was 2.07 ± 0.86 (mean ± SD).

**Table 1:** Characteristics of the patients

Characteristic	Value (n = 20 patients)
Age, years	65 (57–75)
Sex, n (male/female)	12/8
Simplified acute physiology score II	55 (47–59)
Sequential organ failure assessment	9 (7–10)
Causes of intensive care admission:	
Septic shock, n (%)	8 (40 %)
Traumatic brain injury, n (%)	7 (35 %)
Severe trauma, n (%)	3 (15 %)
Hemorrhagic shock, n (%)	1 (5 %)
Cardiac arrest, n (%)	1 (5 %)
Vasopressor dose, μg/kg/min	0.5 (0.0–1.4)
Renal support, n (%)	7 (35 %)
Mechanical ventilation, n (%)	20 (100 %)
Length of mechanical ventilation, days	8 (5–13)
Length of stay in ICU, days	16 (6–22)
ICU death, n (%)	7 (35 %)

Values are presented as median (25th–75th percentile); categorical data are presented as number (percentage)

**Table 2:** Hemodynamic data of the patients

	Value (n = 20 patients)
Heart rate, bpm	97 (82–111)
Mean arterial pressure, mmHg	81 (69–90)
Cardiac index, l.min^−1^.m^−2^	3.3 (2.5–4.)]
Delta pulse pressure, %	10 (7–14)
Lactate, mEq. L^−1^	2.1 (1.2–3.0)

Values are presented as median (25th–75th percentile)

**Fig. 2:**
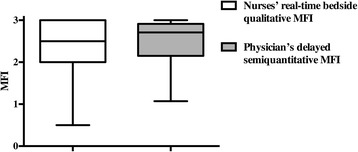
Nurses’ real-time bedside qualitative evaluations of mean fluorescence intensity (*MFI*) vs physician’s delayed semiquantitative MFI evaluation

**Fig. 3:**
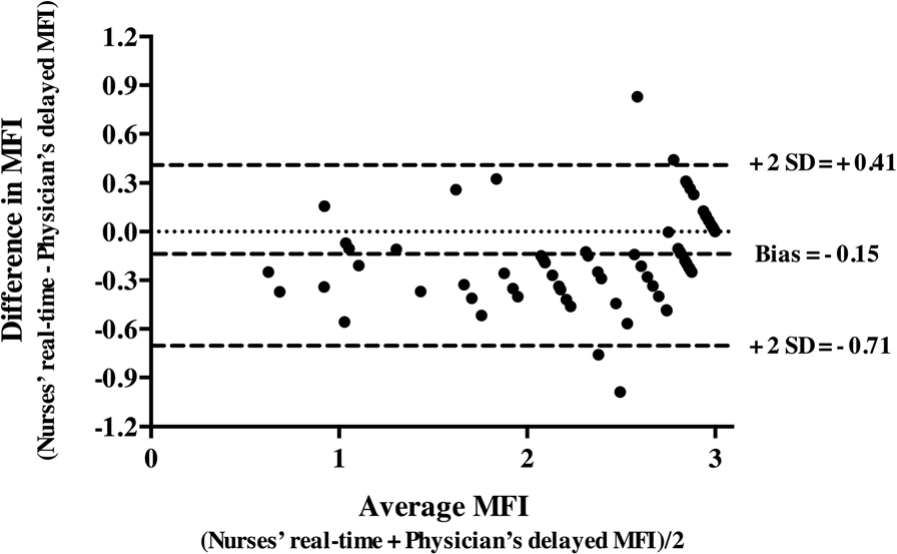
Bland-Altman plot showing the limits of agreement (bias ± 2 SD) between paired values for the nurses’ real-time bedside qualitative mean fluorescence intensity (*MFI*) evaluations and the physician’s delayed semiquantitative MFI evaluation

There were no significant differences between the nurses’ real-time and the physician’s delayed MFI evaluations (Fig. 2). In fact these two values were strongly correlated (*R*^2^ = 0.84, *p* <0.0001). Comparisons made using the Bland–Altman method showed good agreement between the nurses’ real-time MFI and the physician’s delayed MFI (Fig. 3). The mean difference between the two MFIs was −0.15, SD = 0.28 (Fig. 1). On analysis of the potential for heterogeneous performance among nurses, we observed no differences between nurses. The nurses’ real-time MFI assessments had 97 % sensitivity (95 % CI 84, 99 %) and 95 % specificity (95 % CI 84, 99 %) for detecting a delayed MFI <2.5. For density, the median delayed TVD measured by the physician was 10.3 (8.9–12.2) mm/mm^2^. Eighty-one percent of the paramedical qualitative density measurements corresponded with the automatized delayed TVD measurements. The nurses’ real-time TVD assessments had 77 % sensitivity (95 % CI 46, 95 %) and 100 % specificity (95 % CI 89, 100 %) to detect a TVD <8 mm/mm^2^.

## Discussion

The main finding of this study is that a real-time bedside qualitative evaluation of MFI, feasible for performance by nurses, had good agreement with conventional delayed analysis by a physician, and had high sensitivity and specificity at detecting impaired microvascular flow and low capillary density.

Microcirculatory dysfunction is a central abnormality in shock and represents a logical and promising therapeutic target. Several studies, especially in septic shock patients, have reported that macrovascular hemodynamic optimization using therapies such as fluid administration, RBC transfusion or catecholamine infusion could have different impacts on microcirculatory parameters [1, 4, 17–19]. Thus, defining the adequacy of resuscitation requires attention to both macrocirculation and microcirculation. However, to do this, physicians need a reliable and a relevant technique for assessing microcirculation for routine clinical application at the bedside. De backer et al. demonstrated the relevance of sublingual microvascular parameters in terms of outcome [4]. In addition, these parameters are dynamic and are highly sensitive to systemic hemodynamic interventions such as fluid resuscitation, vasopressors or transfusion [1, 4, 17–19].

While visualization of the sublingual circulation has improved in terms of image quality and speed of acquisition and analysis, the use of this technique is still limited in its routine application at the bedside, by its perceived complexity and offline analysis. Evolution of this technology requires the demonstration of the feasibility of data acquisition and analysis at the bedside by medical or paramedical staff, and their ability to guide the management of patient hemodynamics. In this context, the results of the present study are a major step towards rendering accessible this technique at the bedside.

Our results for the analysis of microvascular flow confirm those of Arnold et al. showing that SDF videomicroscopy of the sublingual microcirculation can be used to generate real-time bedside data on microvascular perfusion [20]. These authors found good agreement with conventional offline analysis of MFI (mean difference of −0.031, SD = 0.198 (95 % CI −0.43, 0.37)), and MFI bedside determination was highly sensitive (94 %) and specific (92 %) for detecting impaired microvascular flow. The agreement reported by Arnold et al. is slightly better than that in our present study; however, only one single operator performed the sublingual analysis for their entire cohort of patients. One particularity of our study is the prior training of a large number of nurses to perform microvascular monitoring. On the one hand, having this large number of nurses would be expected to increase inter-individual differences and lead to some bias; on the other hand, this group of nurses could allow us to perform clinical trials of therapies targeting impaired microcirculatory perfusion in ICU patients. Despite the bedside analysis being performed by several nurses, it should be noted that the real-time MFI assessment was excellent. This point is crucial in view of the potential regular use of this technique to alert physicians to the development of low microvascular flow based on the real-time MFI assessment.

For capillary density measurement, Bezemer et al. demonstrated that a rapid automatic assessment of microvascular density in SDF images is possible [12]. However, although their proposed method permitted a statistical gain in time to analyze the density compared with the previous technology (<30 s versus 10 minutes), software analysis was required which would be too difficult at the bedside to allow its real-time application. In this context, we decided to stratify density into three categories (poor, normal and rich). From previous studies comparing sublingual density in critically ill patients, this stratification appears suitable by reflecting patient outcome, severity of illness or efficiency of various therapies such as transfusion, fluid challenge or catecholamine infusion [4, 21]. We found this paramedical evaluation to be simple and quick to perform at the bedside, and found that the visual qualitative assessment of density correlated well with the automatic software measurement, offering good sensitivity and specificity for detecting low density (TVD <8 mm/mm^2^).

As previously mentioned, the fact that the bedside evaluations of MFI and TVD were highly sensitive and specific for detecting impaired microvascular flow and low capillary density suggests that this real-time technique could become part of the nursing supervision. As soon as an impairment of sublingual microcirculation is recognized by nurses, the physician may guide resuscitation to optimize sublingual microcirculation.

We acknowledge some limitations. First, the study included image acquisition only by nurses (images acquired by nurses were reviewed, but images acquired at the same time by physicians were not available). However, the fact that the quality of the nurses’ videos was excellent reasonably excludes the possibility that nurse image acquisition did include a bias. Second, we have only evaluated the TVD and not the perfused vessel density (PVD), which seems to be a better reflection of functional network density [14]. However, considering the need for an efficient real-time bedside analysis, nurses found visual density stratification easy to perform in contrast to the determination of only perfused vessels. Third, it was impossible to measure other microvascular parameters with this bedside analysis, such as the proportion of perfused vessels or the heterogeneity index. However, according to recommendations, the evaluation of one index of capillary density and one index of capillary flow seems fitting in a first approach to analyze the microcirculation network [14]. Fourth, these very encouraging results must be confirmed with a larger number of patients and measurements.

## Conclusion

A real-time bedside qualitative evaluation of MFI and TVD by nurses had good agreement with conventional delayed analysis by a physician, and was highly sensitive and specific for detecting impaired microvascular flow and low capillary density. These results suggest that this real-time technique could become part of the usual surveillance performed by ICU nurses and could be implemented into hemodynamic algorithms in future clinical trials and in regular practice. These results are an essential step to demonstrate whether these real-time measurements have a clinical impact in the management of ICU patients.

## Key messages

- A real-time bedside qualitative evaluation of sublingual microcirculation by nurses had good agreement with conventional delayed analysis by a physician
- A real-time bedside qualitative evaluation of sublingual microcirculation by nurses was highly sensitive and specific for detecting impaired microvascular flow and low capillary density
- A real-time bedside qualitative evaluation of sublingual microcirculation could become part of the usual surveillance performed by ICU nurses and could be implemented into hemodynamic algorithms in future clinical trials and in regular practice
- These results are an essential step to demonstrate whether these real-time measurements have a clinical impact in the management of ICU patients

## Abbreviations

ICU: Intensive care unit
MFI: Microvascular flow index
PPV: Proportion of perfused vessels
RBC: Red blood cells
SAPSII: The simplified acute physiology score II
SDF: Sidestream dark field
SOFA: Sequential organ failure assessment
TVD: Total vessel density
ΔPP: Delta pulse pressure

## References

[1] Trzeciak S, McCoy JV, Phillip Dellinger R, Arnold RC, Rizzuto M, Abate NL (2008). Early increases in microcirculatory perfusion during protocol-directed resuscitation are associated with reduced multi-organ failure at 24 h in patients with sepsis. Intensive Care Med..

[2] De Backer D, Creteur J, Preiser JC, Dubois MJ, Vincent JL (2002). Microvascular blood flow is altered in patients with sepsis. Am J Respir Crit Care Med..

[3] Sakr Y, Dubois MJ, De Backer D, Creteur J, Vincent JL (2004). Persistent microcirculatory alterations are associated with organ failure and death in patients with septic shock. Crit Care Med..

[4] De Backer D, Donadello K, Sakr Y, Ospina-Tascon G, Salgado D, Scolletta S (2013). Microcirculatory alterations in patients with severe sepsis: impact of time of assessment and relationship with outcome. Crit Care Med..

[5] Ince C (2005). The microcirculation is the motor of sepsis. Crit Care..

[6] Harrois A, Dupic L, Duranteau J (2011). Targeting the microcirculation in resuscitation of acutely unwell patients. Curr Opin Crit Care..

[7] Bateman RM, Walley KR (2005). Microvascular resuscitation as a therapeutic goal in severe sepsis. Crit Care..

[8] Nencioni A, Trzeciak S, Shapiro NI (2009). The microcirculation as a diagnostic and therapeutic target in sepsis. Intern Emerg Med..

[9] Trzeciak S, Dellinger RP, Parrillo JE, Guglielmi M, Bajaj J, Abate NL (2007). Early microcirculatory perfusion derangements in patients with severe sepsis and septic shock: relationship to hemodynamics, oxygen transport, and survival. Ann Emerg Med.

[10] Tachon G, Harrois A, Tanaka S, Kato H, Huet O, Pottecher J (2014). Microcirculatory alterations in traumatic hemorrhagic shock. Crit Care Med..

[11] De Backer D, Ospina-Tascon G, Salgado D, Favory R, Creteur J, Vincent JL (2010). Monitoring the microcirculation in the critically ill patient: current methods and future approaches. Intensive Care Med..

[12] Bezemer R, Dobbe JG, Bartels SA, Boerma EC, Elbers PW, Heger M (2011). Rapid automatic assessment of microvascular density in sidestream dark field images. Med Biol Eng Comput..

[13] Aykut GV, Veenstra G, Scorcella C, Ince C, Boerma C (2015). Cytocam-IDF (incident dark field illumination) imaging for bedside monitoring of the microcirculation. Intensive Care Med Exp..

[14] De Backer D, Hollenberg S, Boerma C, Goedhart P, Buchele G, Ospina-Tascon G, et al. How to evaluate the microcirculation: report of a round table conference. Crit Care. 2007;11:R101. http://www.MicroscanAnalysis.blogspot.com Accessed 2007. 10.1186/cc6118 PMC2556744 17845716

[15] Massey MJ, Larochelle E, Najarro G, Karmacharla A, Arnold R, Trzeciak S (2013). The microcirculation image quality score: development and preliminary evaluation of a proposed approach to grading quality of image acquisition for bedside videomicroscopy. J Crit Care..

[16] Bland JM, Altman DG (1986). Statistical methods for assessing agreement between two methods of clinical measurement. Lancet..

[17] Yuruk K, Bartels SA, Milstein DM, Bezemer R, Biemond BJ, Ince C (2012). Red blood cell transfusions and tissue oxygenation in anemic hematology outpatients. Transfusion..

[18] De Backer D, Creteur J, Dubois MJ, Sakr Y, Koch M, Verdant C (2006). The effects of dobutamine on microcirculatory alterations in patients with septic shock are independent of its systemic effects. Crit Care Med..

[19] Pottecher J, Deruddre S, Teboul JL, Georger JF, Laplace C, Benhamou D (2010). Both passive leg raising and intravascular volume expansion improve sublingual microcirculatory perfusion in severe sepsis and septic shock patients. Intensive Care Med..

[20] Arnold RC, Parrillo JE, Phillip Dellinger R, Chansky ME, Shapiro NI, Lundy DJ (2009). Point-of-care assessment of microvascular blood flow in critically ill patients. Intensive Care Med..

[21] Genzel-Boroviczeny O, Christ F, Glas V (2004). Blood transfusion increases functional capillary density in the skin of anemic preterm infants. Pediatr Res..

